# Social Risk Factor Domains and Preventive Care Services in US Adults

**DOI:** 10.1001/jamanetworkopen.2024.37492

**Published:** 2024-10-04

**Authors:** Tamara Schroeder, Mukoso N. Ozieh, Abigail Thorgerson, Joni S. Williams, Rebekah J. Walker, Leonard E. Egede

**Affiliations:** 1 Department of Surgery, University of California, Davis, Sacramento; 2 Division of Nephrology, Department of Medicine, Medical College of Wisconsin, Milwaukee; 3 Center for Advancing Population Science, Medical College of Wisconsin, Milwaukee; 4 Division of Nephrology, Clement J. Zablocki Veterans Affairs Medical Center, Milwaukee, Wisconsin; 5 Division of General Internal Medicine, Department of Medicine, Medical College of Wisconsin, Milwaukee; 6 Department of Medicine, Jacobs School of Medicine and Biomedical Sciences, University at Buffalo, Buffalo, New York

## Abstract

**Importance:**

Growing evidence suggests that social determinants of health are associated with low uptake of preventive care services.

**Objective:**

To examine the independent associations of social risk factor domains with preventive care services among US adults.

**Design, Setting, and Participants:**

This cross-sectional study used National Health Interview Survey data on 82 432 unweighted individuals (239 055 950 weighted) from 2016 to 2018. Subpopulations were created for each of the primary outcomes: routine mammography (women aged 40-74 years), Papanicolaou test (women aged 21-65 years), colonoscopy (adults aged 45-75 years), influenza vaccine (adults aged ≥18 years), and pneumococcal vaccine (adults aged ≥65 years). Statistical analysis was performed from July to December 2023.

**Exposures:**

Six social risk domains (economic instability, lack of community, education deficit, food insecurity, social isolation, and lack of access to care) and a count of domains.

**Main Outcomes and Measures:**

Logistic regression models were used to examine the independent association between each primary outcome (mammography, Papanicolaou test, colonoscopy, influenza vaccine, and pneumococcal vaccine) and social risk factor domains, while controlling for covariates (age, sex, race and ethnicity, health insurance, and comorbidities).

**Results:**

A total of 82 432 unweighted US individuals (239 055 950 weighted individuals) were analyzed. A total of 54.3% were younger than 50 years, and 51.7% were female. All 5 screening outcomes were associated with educational deficit (mammography: odds ratio [OR], 0.73 [95% CI, 0.67-0.80]; Papanicolaou test: OR, 0.78 [95% CI, 0.72-0.85]; influenza vaccine: OR, 0.71 [95% CI, 0.67-0.74]; pneumococcal vaccine: OR, 0.68 [95% CI, 0.63-0.75]; colonoscopy: OR, 0.82 [95% CI, 0.77-0.87]) and a lack of access to care (mammography: OR, 0.32 [95% CI, 0.27-0.38]; Papanicolaou test: OR, 0.49 [95% CI, 0.44-0.54]; influenza vaccine: OR, 0.44 [95% CI, 0.41-0.47]; pneumococcal vaccine: OR, 0.30 [95% CI, 0.25-0.38]; colonoscopy: OR, 0.35 [95% CI, 0.30-0.41]). Fully adjusted models showed that every unit increase in social risk count was significantly associated with decreased odds of receiving a mammography (OR, 0.74 [95% CI, 0.71-0.77]), Papanicolaou test (OR, 0.84 [95% CI, 0.81-0.87]), influenza vaccine (OR, 0.81 [95% CI, 0.80-0.83]), pneumococcal vaccine (OR, 0.80 [95% CI, 0.77-0.83]), and colonoscopy (OR, 0.88 [95% CI, 0.86-0.90]).

**Conclusions and Relevance:**

This cross-sectional study of US adults suggests that social risk factor domains were associated with decreased odds of receiving preventive services; this association was cumulative. There is a need to address social risk factors to optimize receipt of recommended preventive services.

## Key Points

**Question:**

What is the independent association of social risk factor domains with receipt of preventive care services among US adults?

**Findings:**

In this cross-sectional study of 82 432 unweighted US adults (239 055 950 weighted), social risk factor domains were independently associated with decreased odds of receiving preventive care services; this association was cumulative, where each additional social risk domain was associated with decreased odds of receiving preventive care services. The domains of educational deficit and the lack of access to care were associated with decreased odds of receiving all preventive care services examined in this study.

**Meaning:**

This study suggests that there is a need to address social risk factors to optimize receipt of recommended preventive services; future prospective research studies are needed to investigate the underlying mechanisms of these associations.

## Teaser

This cross-sectional study examines the independent associations of social risk factor domains with odds of receiving preventive care services among US adults.

## Introduction

Preventive care services, including vaccinations and cancer screenings, effectively reduce the burden of vaccine-preventable diseases, cancer morbidity, and mortality.^1,2^ It is estimated that the number of life-years gained from cancer screenings ranges from 12 million to 16 million people, which translates to an aggregate value of at least $7 trillion.^3^ The Centers for Disease Control and Prevention (CDC) and the United States Preventive Services Task Force (USPSTF) recommend influenza and pneumococcal vaccinations and cancer screenings, including breast, cervical, and colorectal, for adults, based on age and health conditions.^4,5,6^ However, evidence shows that only approximately 8% of adults receive all recommended preventive care services.^7^

Growing evidence suggests that social determinants of health are associated with the low uptake of preventive care services.^8,9,10,11^ Social determinants of health are the nonmedical conditions in which people are born, live, work, and age that shape health for better or worse.^11^ Social risk factors, on the other hand, are specific adverse social conditions, such as low educational level, housing instability, and social isolation, that are associated with poor health and health outcomes, including cancer outcomes.^8,11,12^ Few studies have examined the individual associations between social risk factors and preventive care services, specifically looking at the independent associations of social risk factor domains and preventive care services.

Therefore, the main aim of this study was to examine the independent associations of social risk factor domains with preventive care services among US adults. Using the Kaiser Family Foundation framework, there are 6 social risk factor domains: (1) economy instability, (2) education, (3) community and social context, (4) neighborhood and physical environment, (5) health care system, and (6) food environment.^13^ We hypothesized that social risk domains would be independently associated with a lower likelihood of receiving recommended age-appropriate clinical preventive care services.

## Methods

The Medical College of Wisconsin did not require approval for this cross-sectional study because the data used are publicly accessible and anonymized. Reporting followed the Strengthening the Reporting of Observational Studies in Epidemiology (STROBE) reporting guideline.

### Data Source and Population

The National Health Interview Survey (NHIS) is a national survey that gathers information regarding health and health-related matters for the past 60 years. The NHIS contains disease, food security, and health care information among other data on people living in the US.^14^ The primary aim of the NHIS is to oversee the well-being of the US population by gathering and examining information on a wide array of health-related subjects. The data are collected with a combination of computer surveys, in-person surveys, and telephone surveys.

We analyzed 2016-2018 data from the adult, person, and family files of the NHIS. The main sample consisted of 82 432 US adults aged 18 years or older, representing a weighted sample of 239 055 950 noninstitutionalized US adults. Subpopulations were created from the main sample based on the USPSTF and CDC guidelines on the target population for medical screenings.^2,4^ The target populations included those recommended to receive mammography (women aged 40-74 years: 25 064 unweighted and 66 372 158 weighted), Papanicolaou test (women aged 21-65 years: 30 911 unweighted and 92 033 502 weighted), a colonoscopy (adults aged 45-75 years: 41 690 unweighted and 110 944 064 weighted), influenza vaccine (adults aged ≥18 years: 82 171 unweighted and 238 152 642 weighted), and pneumococcal vaccine (adults aged ≥65 years: 22 397 unweighted and 47 204 126 weighted). The subpopulation samples excluded those missing the screening information, including those who felt uncomfortable answering or those who did not know the answer to a question.

### Primary Outcomes

Five age-appropriate self-reported preventive care services included receipt of (1) mammography, (2) Papanicolaou test, (3) colonoscopy, (4) influenza vaccine, and (5) pneumococcal vaccine. These outcomes were selected based on a universal practice agreement for screening guidelines. The questions regarding mammography, Papanicolaou test, colonoscopy, and influenza vaccine screenings asked for use of these services in the past 12 months. The question regarding the pneumococcal vaccine asked if the vaccine was ever used.

### Primary Independent Variables

Six social risk domains included (1) economic instability, (2) educational deficit, (3) lack of community, (4) food insecurity, (5) social isolation, and (6) lack of access to care. Based on prior work, questions asked at the baseline interview were mapped to each of the social risk domains (eTable in Supplement 1).^15^ Each social risk domain was coded as “yes” to any one of the items in a domain and “no” for no to all items in a domain. Missing was defined as completely missing all items in a domain. An indicator variable for social risk factor domain count was then created, counting the number of domains an individual indicated “yes,” ranging from 0 to 6 (where 0 indicates no social risk domains and 6 indicates 6 social risk domains).

### Covariates

The covariates included were age (18-39 years, 40-49 years, 50-64 years, 65-74 years, and ≥75 years), race and ethnicity (Hispanic, non-Hispanic Black, non-Hispanic White, and non-Hispanic other [American Indian or Alaska Native, Asian, and multiple races]), health insurance status (yes or no), and comorbidities (obesity, mental illness, hypertension, coronary heart disease, myocardial infarction, stroke, asthma, ulcer, cancer, emphysema or chronic obstructive pulmonary disease, kidney disease, diabetes, liver disease, arthritis, migraine, and chronic pain). The individual comorbidities were further categorized as a count, with each individual having 0, 1 to 2, 3 to 4, or 5 or more self-reported comorbidities. Race and ethnicity was self-reported; the categories were created from variables within the NHIS dataset. Race and ethnicity is a social construct, but is included as a covariate because of the possible explanatory strength in the association of interest.

### Statistical Analysis

Statistical analysis was performed from July to December 2023. Descriptive analyses were reported for each variable as a percentage. No comparisons or other testing were done in the descriptive tables. Logistic regression models were created for each outcome and its corresponding population: mammography, Papanicolaou test, colonoscopy, influenza vaccine, and pneumococcal vaccine. The unadjusted models included each of the 6 social risk domains separately. Models were then run with all 6 domains in the same model. Last, the models were adjusted for all covariates, including age, race and ethnicity, insurance status, individual comorbidities, and the survey year. Logistic regression models were also run on the same outcomes with the social risk factor domains as a count (0-6) used as the primary independent variable. These models were also adjusted for covariates and survey year. R, version 4.0.3 (R Project for Statistical Computing) was used for the analysis, *P* values were from 2-sided tests, and *P* < .05 was determined to be statistically significant. All analyses were weighted using the svydesign function from the survey package in R according to NHIS information.

## Results

Sample demographics for 82 432 unweighted (239 055 950 weighted) US adults aged 18 years or older who answered questions regarding social risk factors and preventive care services through the NHIS between 2016 and 2018 are presented in Table 1. Just over half the sample was younger than 50 years (54.3%); 51.7% of adults were female, and 48.2% were male. A total of 16.1% of the sample of US adults were Hispanic, 12.0% were non-Hispanic Black, 64.8% were non-Hispanic White, and 7.2% were non-Hispanic Other race or ethnicity. Most of the sample had health insurance (90.6%) and reported having 1 or more comorbid disease (63.5%).

**Table 1.** Characteristics of Adults Aged 18 Years or Older, National Health Interview Survey, 2016-2018

Characteristic	Total sample (N = 82 432)^a^		2016 (n = 31 928)		2017 (n = 25 791)		2018 (n = 24 713)	
	Weighted %	No. (%)	Weighted %	No. (%)	Weighted %	No. (%)	Weighted %	No. (%)
Demographic								
Age, y								
18-39	38.0	25 998 (31.5)	38.1	10 251 (32.1)	38.2	8250 (32.0)	37.6	7497 (30.3)
40-49	16.3	11 975 (14.5)	16.3	4579 (14.3)	16.0	3718 (14.4)	16.4	3678 (14.9)
50-64	25.6	21 582 (26.2)	25.9	8442 (26.4)	25.	6734 (26.1)	25.2	6406 (25.9)
65-74	12.0	13 231 (16.1)	11.7	4973 (15.6)	12.0	4098 (15.9)	12.4	4160 (16.8)
≥75	8.1	9646 (11.7)	8.0	3683 (11.5)	8.1	2991 (11.6)	8.3	2972 (12.0)
Sex								
Male	48.3	37 440 (45.4)	48.3	14 509 (45.4)	48.3	11 668 (45.2)	48.4	11 263 (45.6)
Female	51.7	44 992 (54.6)	51.7	17 419 (54.6)	51.7	14 123 (54.8)	51.6	13 450 (54.4)
Race and ethnicity								
Hispanic	16.1	9883 (12.0)	15.8	3673 (11.5)	16.0	3122 (12.1)	16.4	3088 (12.5)
Non-Hispanic Black	12.0	9093 (11.0)	11.9	3456 (10.8)	12.0	2806 (10.9)	12.0	2831 (11.5)
Non-Hispanic White	64.8	58 090 (70.5)	65.3	22 683 (71.0)	64.9	18 244 (70.7)	64.1	17 163 (69.4)
Non-Hispanic other^b^	7.2	5366 (6.5)	7.0	2116 (6.6)	7.1	1619 (6.3)	7.5	1631 (6.6)
Health insurance	90.6	75 272 (91.7)	91.0	29 221 (91.9)	90.5	23 548 (91.6)	90.3	22 503 (91.3)
Comorbidities								
Obesity^c^	30.8	24 750 (30.8)	30.0	9396 (30.2)	30.9	7814 (31.2)	31.5	7540 (31.3)
Mental health issue^d^	2.7	2414 (2.9)	2.4	886 (2.8)	2.5	718 (2.8)	3.1	810 (3.3)
Hypertension	31.1	29 363 (35.7)	31.3	11 328 (35.5)	30.6	9070 (35.2)	31.5	8965 (36.3)
Coronary heart disease	4.5	4582 (5.6)	4.5	1764 (5.5)	4.4	1365 (5.3)	4.6	1453 (5.9)
Heart attack	3.1	3249 (4.0)	3.1	1253 (3.9)	3.0	981 (3.8)	3.1	1015 (4.1)
Stroke	3.1	3121 (4.0)	3.1	1187 (3.7)	3.1	968 (3.8)	3.1	966 (3.9)
Asthma	13.6	11 333 (13.8)	13.9	4374 (13.7)	13.5	3605 (14.0)	13.4	3354 (13.6)
Ulcer	6.1	5807 (7.1)	6.0	2237 (7.0)	6.2	1815 (7.0)	6.0	1755 (7.1)
Cancer	9.4	9437 (11.5)	9.4	3590 (11.3)	9.5	2973 (11.5)	9.4	2874 (11.6)
Emphysema or COPD	3.7	4000 (4.9)	3.6	1525 (4.8)	3.8	1239 (4.8)	3.9	1236 (5.0)
Kidney disease	2.2	2127 (2.6)	2.0	774 (2.4)	2.1	654 (2.5)	2.4	699 (2.8)
Diabetes	9.9	8975 (11.2)	9.7	3408 (11.0)	9.6	2709 (10.8)	10.4	2858 (11.9)
Liver disease	1.9	1646 (2.0)	2.0	653 (2.0)	1.8	510 (2.0)	1.8	483 (2.0)
Arthritis	23.8	23 256 (28.3)	23.8	8927 (28.0)	23.8	7313 (28.4)	23.8	7016 (28.4)
Migraine	15.2	12 454 (15.1)	15.0	4828 (15.1)	15.1	3862 (15.0)	15.5	3764 (15.2)
Chronic pain	34.3	31 377 (38.1)	34.0	12 126 (38.0)	34.4	9814 (38.1)	34.4	9437 (38.2)
Comorbidity count								
0	33.0	23 601 (28.6)	33.2	9282 (29.1)	33.1	7342 (28.5)	32.8	6977 (28.2)
1-2	39.7	32 663 (39.6)	39.6	12 595 (39.4)	39.8	10 303 (39.9)	39.8	9765 (39.5)
3-4	17.1	16 217 (19.7)	17.3	6260 (19.6)	17.1	5080 (19.7)	17.0	4877 (19.7)
≥5	6.7	6968 (8.5)	6.7	2691 (8.4)	6.5	2107 (8.2)	7.0	2170 (8.8)
Survey year								
2016	33.0	31 928 (38.7)	NA	NA	NA	NA	NA	NA
2017	33.2	25 791 (31.3)	NA	NA	NA	NA	NA	NA
2018	33.8	24 713 (30.0)	NA	NA	NA	NA	NA	NA

Abbreviations: COPD: chronic obstructive pulmonary disease; NA, not applicable.

^a^ N = 82 432 unweighted participants, representing 239 055 950 weighed participants.

^b^ American Indian or Alaska Native, Asian, and multiple races.

^c^ Obesity was defined as having a body mass index (calculated as weight in kilograms divided by height in meters squared) of 30 or greater.

^d^ Mental health issue was defined as reporting “depression/anxiety/emotional problem causes difficulty with activity.”

The prevalence of receiving preventive care services by social risk domain is shown in Table 2. A total of 68.6% of women aged 40 to 74 years completed a screening mammography, and 66.6% of women aged 21 to 65 years completed a Papanicolaou test. More than one-third of US adults aged 45 to 75 years (35.0%) received a screening colonoscopy, 43.5% of US adults aged 18 years or older received the influenza vaccine, and 68.4% of US adults aged 65 years or older received the pneumococcal vaccine. Individuals with a lack of access to care had the lowest prevalence of receiving all recommended screenings, with 33.2% reporting pneumococcal vaccine receipt, 13.2% reporting colonoscopy receipt, 18.1% reporting influenza vaccine receipt, 37.6% reporting mammograpy receipt, and 50.9% reporting Papanicolaou test receipt. Individuals with food insecurity and educational deficit had the next 2 lowest levels of receiving all recommended preventive health care screenings.

**Table 2.** Prevalence of Receipt of Preventive Care Services by Social Risk Domains, National Health Interview Survey, 2016-2018^a^

Social risk domain	Mammography, %	Papanicolaou test, %	Colonoscopy, %	Vaccine, %	
				Influenza	Pneumococcal
Overall prevalence	68.6	66.6	35.0	43.5	68.4
Economic instability	67.6	65.5	35.4	44.4	69.3
Lack of community	65.1	64.8	34.1	40.3	67.6
Educational deficit	63.0	60.7	31.4	38.4	63.7
Food insecurity	58.8	62.7	30.9	35.3	58.9
Social isolation	66.7	62.3	37.6	48.2	69.2
Lack access to care	37.6	50.9	13.2	18.1	33.2

^a^ Reported percentages are weighted.

Table 3 shows logistic regression models for receiving a screening mammography, Papanicolaou test, colonoscopy, influenza vaccine, and pneumococcal vaccine, adjusting for all 6 social risk domains and relevant covariates. All 5 screening outcomes were associated with educational deficit (mammography: odds ratio [OR], 0.73 [95% CI, 0.67-0.80]; Papanicolaou test: OR, 0.78 [95% CI, 0.72-0.85]; influenza vaccine: OR, 0.71 [95% CI, 0.67-0.74]; pneumococcal vaccine: OR, 0.68 [95% CI, 0.63-0.75]; colonoscopy: OR, 0.82 [95% CI, 0.77-0.87]) and lack of access to care (mammography: OR, 0.32 [95% CI, 0.27-0.38]; Papanicolaou test: OR, 0.49 [95% CI, 0.44-0.54]; influenza vaccine: OR, 0.44 [95% CI, 0.41-0.47]; pneumococcal vaccine: OR, 0.30 [95% CI, 0.25-0.38]; colonoscopy: OR, 0.35 [95% CI, 0.30-0.41]). All social risk domains were associated with decreased odds of receiving a screening mammography and the influenza vaccine. Lack of community (OR, 0.92 [95% CI, 0.85-0.99]), educational deficit (OR, 0.78 [95% CI, 0.72-0.85]), social isolation (OR, 0.80 [95% CI, 0.75-0.87]), and lack of access to care (OR, 0.49 [95%, CI 0.44-0.54]) were associated with decreased odds of receiving a Papanicolaou test. This finding means that, for example, social isolation was associated with 20% lower odds of receiving a Papanicolaou test, and lack of access to care was associated with 51% lower odds of receiving a Papanicolaou test. However, economic instability (OR, 0.96 [95%, CI 0.88-1.04]) and food insecurity (OR, 0.93 [95% CI, 0.85-1.01]) domains were not significantly associated with receiving a Papanicolaou test. All 6 social risk domains except lack of community were significantly associated with receiving a pneumococcal vaccine. After adjustment for all 6 social risk domain variables and covariates, only educational deficit (OR, 0.82 [95% CI, 0.77-0.87]), food insecurity (OR, 0.82 [95% CI 0.75-0.90]), and lack access to care (OR, 0.35 [95% CI, 0.30-0.41]) were significantly associated with decreased odds of receiving a colonoscopy.

**Table 3.** Logistic Regression Models for Receiving Preventive Care Services, National Health Interview Survey, 2016-2018

Characteristic	Odds ratio (95% CI)		
	Unadjusted	Domain adjusted^a^	Fully adjusted^b^
**Mammography**			
Economic instability	0.79 (0.73-0.86)^c^	0.92 (0.85-1.01)	0.87 (0.78-0.98)^d^
Lack of community	0.77 (0.72-0.83)^c^	0.86 (0.80-0.93)^c^	0.84 (0.77-0.92)^c^
Educational deficit	0.67 (0.62-0.72)^c^	0.79 (0.73-0.85)^c^	0.73 (0.67-0.80)^c^
Food insecurity	0.57 (0.53-0.62)^c^	0.67 (0.61-0.73)^c^	0.70 (0.63-0.79)^c^
Social isolation	0.88 (0.82-0.94)^c^	0.98 (0.91-1.05)	0.76 (0.70-0.83)^c^
Lack of access to care	0.25 (0.21-0.29)^c^	0.27 (0.23-0.32)^c^	0.32 (0.27-0.38)^c^
**Papanicolaou test**			
Economic instability	0.81 (0.75-0.86)^c^	0.89 (0.83-0.96)^e^	0.96 (0.88-1.04)
Lack of community	0.87 (0.81-0.93)^c^	0.95 (0.88-1.02)	0.92 (0.85-0.99)^d^
Educational deficit	0.68 (0.64-0.73)^c^	0.73 (0.68-0.78)^c^	0.78 (0.72-0.85)^c^
Food insecurity	0.78 (0.73-0.84)^c^	0.95 (0.89-1.02)	0.93 (0.85-1.01)
Social isolation	0.78 (0.73-0.83)^c^	0.80 (0.75-0.85)^c^	0.80 (0.75-0.87)^c^
Lack of access to care	0.47 (0.43-0.52)^c^	0.49 (0.45-0.54)^c^	0.49 (0.44-0.54)^c^
**Influenza vaccine**			
Economic instability	1.16 (1.11-1.21)^c^	1.22 (1.17-1.28)^c^	0.92 (0.87-0.96)^c^
Lack of community	0.81 (0.78-0.84)^c^	0.88 (0.84-0.91)^c^	0.92 (0.88-0.96)^c^
Educational deficit	0.71 (0.69-0.74)^c^	0.78 (0.75-0.81)^c^	0.71 (0.67-0.74)^c^
Food insecurity	0.64 (0.61-0.67)^c^	0.69 (0.66-0.73)^c^	0.84 (0.80-0.89)^c^
Social isolation	1.30 (1.25-1.35)^c^	1.33 (1.28-1.39)^c^	0.94 (0.89-0.98)^e^
Lack of access to care	0.25 (0.23-0.26)^c^	0.26 (0.25-0.28)^c^	0.44 (0.41-0.47)^c^
**Pneumococcal vaccine**			
Economic instability	1.50 (1.35-1.66)^c^	1.65 (1.47-1.84)^c^	1.32 (1.17-1.49)^c^
Lack of community	0.95 (0.87-1.03)	0.98 (0.91-1.07)	0.93 (0.85-1.01)
Educational deficit	0.68 (0.63-0.73)^c^	0.70 (0.64-0.75)^c^	0.68 (0.63-0.75)^c^
Food insecurity	0.61 (0.55-0.68)^c^	0.64 (0.58-0.71)^c^	0.67 (0.60-0.76)^c^
Social isolation	1.06 (0.99-1.14)	1.14 (1.06-1.22)^c^	0.88 (0.81-0.95)^e^
Lack of access to care	0.22 (0.18-0.26)^c^	0.22 (0.18-0.27)^c^	0.30 (0.25-0.38)^c^
**Colonoscopy**			
Economic instability	1.07 (1.00-1.14)^d^	1.11 (1.03-1.18)^e^	0.95 (0.88-1.02)
Lack of community	0.94 (0.89-0.99)^d^	0.97 (0.92-1.02)	0.98 (0.92-1.04)
Educational deficit	0.77 (0.73-0.81)^c^	0.82 (0.77-0.86)^c^	0.82 (0.77-0.87)^c^
Food insecurity	0.79 (0.74-0.85)^c^	0.81 (0.76-0.87)^c^	0.82 (0.75-0.90)^c^
Social isolation	1.18 (1.12-1.24)^c^	1.24 (1.18-1.31)^c^	1.03 (0.97-1.09)
Lack of access to care	0.26 (0.23-0.30)^c^	0.27 (0.24-0.31)^c^	0.35 (0.30-0.41)^c^

^a^ Adjusted for social risk domains only.

^b^ Adjusted for social risk domains, age, sex, race and ethnicity, insurance status, obesity, mental health, comorbidities, and survey year.

^c^ *P* < .001.

^d^ *P* < .05.

^e^ *P* < .01.

Table 4 shows logistic regression models for the association between the cumulative social risk measure and receiving a mammography, Papanicolaou test, colonoscopy, influenza vaccine, and pneumococcal vaccine, adjusting for relevant covariates. Fully adjusted models showed that every unit increase in social risk count was significantly associated with decreased odds of receiving a mammography (OR, 0.74 [95% CI, 0.71-0.77]), Papanicolaou test (OR, 0.84 [95% CI, 0.81-0.87]), influenza vaccine (OR, 0.81 [95% CI, 0.80-0.83]), pneumococcal vaccine (OR, 0.80 [95% CI, 0.77-0.83]), and colonoscopy (OR, 0.88 [95% CI, 0.86-0.90]).

**Table 4.** Logistic Regression Models for Association of Cumulative Social Risk With Receipt of Preventive Care Services, National Health Interview Survey, 2016-2018

Social risk count^a^	Odds ratio (95% CI)	
	Unadjusted	Fully adjusted^b^
Mammography	0.78 (0.76-0.80)^c^	0.74 (0.71-0.77)^c^
Papanicolaou test	0.83 (0.81-0.85)^c^	0.84 (0.81-0.87)^c^
Influenza vaccine	0.86 (0.84-0.87)^c^	0.81 (0.80-0.83)^c^
Pneumococcal vaccine	0.88 (0.85-0.91)^c^	0.80 (0.77-0.83)^c^
Colonoscopy	0.92 (0.90-0.94)^c^	0.88 (0.86-0.90)^c^

^a^ Social risk count ranged from 0 to 6, and odds are for every unit increase in social risk count.

^b^ Adjusted for age, sex, race and ethnicity, insurance status, obesity, mental health, comorbidities, and survey year.

^c^ *P* < .001.

## Discussion

In this cross-sectional study of US adults eligible for preventive health care screening, we found that social risk factor domains were differentially and independently associated with decreased odds of receiving preventive care services recommended by the USPSTF. Specifically, the social risk domains of educational deficit and lack of access to care were associated with decreased odds of receiving all preventive care services examined in this study. Lack of community and social isolation were associated with decreased odds of receiving a screening mammography, Papanicolaou test, and influenza vaccine. Economic instability and food insecurity were associated with decreased odds of receiving a screening mammography, influenza vaccine, and pneumococcal vaccine. In addition, we found that the association between social risk factor domains and receiving recommended preventive care services was cumulative. Each additional social risk domain was associated with decreased odds of receiving preventive care services: 26% for mammography (OR, 0.74 [95% CI, 0.71-0.77]), 16% for Papanicolaou test (OR, 0.84 [95% CI, 0.81-0.87]), 19% for influenza vaccine (OR, 0.81 [95% CI, 0.80-0.83]), 20% for pneumococcal vaccine (OR, 0.80 [95% CI, 0.77-0.83]), and 12% for colonoscopy (OR, 0.88 [95% CI, 0.86-0.90]). These findings suggest that there is a need to address nonmedical factors, especially educational deficit, food insecurity, and lack of access to care, to increase receipt of recommended preventive care services. Our study findings also offer possible intervention targets given the increasing incidence of early-onset cancers among adults younger than 50 years and the persistence of health disparities in cancer incidence in the US.^16^

Our study findings on the association between educational deficit and receiving preventive care services align with prior research. A systematic review and meta-analysis on the association of educational attainment with breast and cervical cancer screening adherence found that the odds of having at least 1 Papanicolaou test over a 3-year period was 96% higher for women with the highest level of education compared with women with the lowest level of education.^17^ They also found that there was a positive association between the level of education and mammography screening irrespective of guidelines followed (1 mammography every 2 years for women aged ≥50 years vs women aged ≥40 years). Educational attainment was defined as health literacy, or “the degree to which individuals and organizations find, understand, and use health-related information or services”; low educational attainment was associated with poor receipt of recommended preventive services.^18,19^ A population-based study examining 3112 women who had either actively declined screening or did not make it to their Papanicolaou test found that women who had actively declined screening felt that the test was of low relevance, while others lacked an understanding of their risk factors.^19^ A major limitation of existing studies and theories examining the association between educational attainment and health outcomes is that they are focused largely on individual-level factors and do not account for the sociopolitical context, including the educational quality, in which education is embedded.^20^ There is a need for a shift in the education-health paradigm from individual education attainment to macrolevel factors associated with educational deficits, such as the quality of education and quality of teachers and educational content, that shape the life course of education. Such a shift will be critical to improving the relationship between patients and their health care professionals, including breakdowns in communication, trust, and perceptions of low quality of care, which have been associated with low use of preventive care services.^7,21,22,23^

Our findings are also consistent with the limited evidence on the association between food insecurity and preventive care services.^24,25,26^ In our study, individuals reporting food insecurity had 11% to 33% lower odds of receiving all 5 recommended screening services. Mendoza et al,^25^ using data from 9 National Cancer Institute (NCI)–designated cancer centers in the US, found that food insecurity was significantly associated with 40% lower odds of being up to date with receiving breast cancer screening and 30% lower odds of receiving colorectal cancer screening even after adjusting for demographic characteristics, health insurance, and financial security. Similarly, another study examining a sample of 2861 women aged between 50 and 74 years residing in the US found that those with food insecurity had 54% lower odds of reporting breast cancer screening compared with those with food security.^26^ The association between food insecurity and health outcomes, including cancer outcomes, is hypothesized to be bidirectional, wherein food insecurity acts as a risk factor for poor health and poor health is a risk factor for food insecurity.^24,27^ Ultimately, food insecurity results in coping strategies, which include a diversion of cognitive attention from health needs to obtaining food.^27^ Evidence suggests that multilevel strategies to address food insecurity, at the household, community, and policy level, are needed to improve health outcomes.^27^ Prospective and longitudinal studies are needed to understand the association of food insecurity with preventive screening outcomes, including diagnosis, treatment, and survival.

Lack of access to care was associated with decreased odds of receiving all preventive care services examined in this study. Access to care has increased in recent years after the enactment of the Patient Protection and Affordable Care Act (ACA), with an approximately 40% decrease in uninsured individuals.^28^ However, evidence suggests that the use of preventive care services remains low, and there is conflicting evidence on the association of the ACA with cancer screening and vaccine uptake. For instance, a 10-year evaluation of the ACA showed that there has been a slight increase in early cancer screenings.^29^ In contrast, Hawks et al^30^ showed that cancer screening, particularly receipt of mammography, has not improved after implementation of the ACA.

Overall, receipt of most preventive screenings in this study was lower among individuals reporting social risk. Compared with prior research,^7,31^ the prevalence of receipt of colonoscopy was lower overall and among individuals reporting social risks. Borsky et al^7^ reported a prevalence of 64% for colon cancer screening in 2015, while the NCI reported a prevalence of 72% for colon cancer screening in 2021.^31^ These observed differences were likely associated with methodological variations. For instance, the NCI included other colon cancer screening approaches, such as stool-based tests (eg, fecal occult blood testing and stool DNA testing) and other optical screening tests (such as computed tomographic colonography and sigmoidoscopy).

Our study findings reaffirm the need for a focus on nonmedical determinants of health. There is a need for multicomponent research interventions that integrate nonmedical and medical determinants of health. In addition, studies are needed to further elucidate and contextualize the association of individual and multidimensional social adversity with receipt of recommended preventive care services. Clinically, there is a need for health care professionals to be aware of the adverse association of social risk factors with receipt of recommended preventive care services and overall health outcomes. Furthermore, clinicians need to be intentional about communicating and providing clear and easy-to-understand information while recognizing that even health-literate patients may struggle with understanding health information when they are afraid and depending on their medical and nonmedical competing needs.^32^ Finally, policymakers need to focus on macrolevel factors that affect the quality of education, such as establishing funding for school systems that is not dependent on the local tax base.^33^

### Strengths and Limitations

Our study has some strengths. Unlike prior studies, our study uniquely examines the independent and cumulative associations of multiple social risk domains with receiving preventive care services. However, there are a few limitations worthy of note. First, due to its cross-sectional study design, we are unable to make causal inferences. Second, participants lacking screening information either due to refusing to answer questions or to not knowing the answer were excluded, which might result in underestimation or overestimation of study estimates. Third, receipt of recommended preventive care services was based on self-report and was not confirmed in the participants’ medical records and, as such, is subject to recall bias. However, evidence shows that self-report of receipt of preventive health care service has good validity.^34^ In addition, the NHIS is a nationally representative dataset used by large epidemiologic studies for decades, including those conducted by the CDC to monitor the health of the nation.^35^

## Conclusions

Overall, the results of this cross-sectional study revealed that specific social risk factor domains were associated with decreased odds of US adults receiving preventive care services. The association between social risk factor domains and receiving recommended preventive care services was cumulative. There is a need to address social risk factors to optimize receipt of recommended preventive services.

## Notes

Supplement 1. **eTable.** Social Determinants of Health Domain Questions in Adults 18 Years and Older, National Health Interview Survey (NHIS), 2016-2018

Supplement 2.
Data Sharing Statement
