# Supplementary material for: Social Risk Factor Domains and Preventive Care Services in US Adults
Source: JAMA Netw Open. 2024 Oct 4;7(10):e2437492. doi: 10.1001/jamanetworkopen.2024.37492 (PMC11452812; doi:10.1001/jamanetworkopen.2024.37492)
Supplement: Supplement 1. — eTable. Social Determinants of Health Domain Questions in Adults 18 Years and Older, National Health Interview Survey (NHIS), 2016-2018 [file jamanetwopen-e2437492-s001.pdf]

# Supplementary Online Content

Schroeder T, Ozieh MN, Thorgerson A, Williams JS, Walker RJ, Egede LE. Social risk factor domains and preventive care services in US adults. *JAMA Netw Open.* 2024;7(10):e2437492. doi:10.1001/jamanetworkopen.2024.37492

**eTable.** Social Determinants of Health Domain Questions in Adults 18 Years and Older, National Health Interview Survey (NHIS), 2016-2018

This supplementary material has been provided by the authors to give readers additional information about their work.

© 2024 Schroeder T et al. *JAMA Network Open.*

**eTable.** Social Determinants of Health Domain Questions in Adults 18 Years and Older, National Health Interview Survey (NHIS), 2016-2018

| | **Total Sample (N = 239,055,950) (n = 82,432)** |
|---|---|
| **Economic instability** | |
| Welfare assistance | 1.2% |
| Income from state/county welfare | 0.8% |
| Unemployed | 37.6% |
| Ever applied for Social Security Disability Insurance (SSDI) | 7.7% |
| Subsidized rent | 3.2% |
| Worry about maintaining current standard of living | 36.1% |
| Worry about enough money for retirement | 44.2% |
| Worry about paying normal monthly bills | 26.5% |
| Worry about inability to pay rent, mortgage, or housing costs | 21.0% |
| Worry about making minimum payment on credit cards | 11.7% |
| **Lack of community** | |
| People in your neighborhood do not help each other out | 16.7% |
| There are no people you can count on in your neighborhood | 17.5% |
| People in your neighborhood cannot be trusted | 16.1% |
| Do not live in a close-knit neighborhood | 35.6% |
| **Educational deficit** | |
| No college or graduate degree | 36.3% |
| English not well spoken | 5.4% |
| **Food insecurity** | |
| Lose weight because not enough money for food | 2.0% |
| Cut size of meals or skip meals in the past month | 5.4% |
| Eat less than you should because not enough money for food | 5.6% |
| Ever hungry but did not eat because no money for food | 3.4% |
| Ever receive food stamps/Supplemental Nutrition Assistance Program (SNAP) in past year | 12.1% |
| Worried that food would run out | 12.6% |
| Food did not last until you could buy more | 10.8% |
| Did not eat balanced meals due to costs | 10.0% |
| Received benefits or food subsidies from WIC program | 4.3% |
| **Social isolation** | |
| Lives alone | 18.0% |
| Difficult to participate in social activities | 8.4% |
| Difficult to going to events | 9.9% |
| Delayed getting medical care due to lack of transportation | 2.0% |
| **Inadequate access to care** | |
| Lacks regular place to go to when sick or need health advice | 13.1% |

© 2024 Schroeder T et al. *JAMA Network Open.*
