# Supplementary material for: Social Risk Factor Domains and Preventive Care Services in US Adults
Source: JAMA Netw Open. 2024 Oct 4;7(10):e2437492. doi: 10.1001/jamanetworkopen.2024.37492 (PMC11452812; doi:10.1001/jamanetworkopen.2024.37492)
Supplement: Supplement 2. — Data Sharing Statement [file jamanetwopen-e2437492-s002.pdf]

Data Sharing Statement

Schroeder. Social Risk Factor Domains and Preventive Care Services in US Adults. *JAMA Netw Open*. Published October 04, 2024. doi:10.1001/jamanetworkopen.2024.37492

Data

Data available: Data available upon reasonable request.
